# Simultaneous Ultra Performance Liquid Chromatography Determination and Antioxidant Activity of Linarin, Luteolin, Chlorogenic Acid and Apigenin in Different Parts of Compositae Species

**DOI:** 10.3390/molecules21111609

**Published:** 2016-11-23

**Authors:** Seung Hwan Hwang, Ji Hun Paek, Soon Sung Lim

**Affiliations:** 1 Department of Food Science and Nutrition, Hallym University, 1 Hallymdeahak-gil, Chuncheon 24252, Korea; isohsh@gmail.com (S.H.H.); hun6678@gmail.com (J.H.P.); 2 Institute of Natural Medicine, Hallym University, 1 Hallymdeahak-gil, Chuncheon 24252, Korea

**Keywords:** UPLC, linarin, luteolin, chlorogenic, apigenin, Compositae

## Abstract

Linarin (LA), luteolin (LE), chlorogenic acid (CA) and apigenin (AP) are four major flavonoids with various promising bioactivities found in Compositae (COP) species. A reliable, reproducible and accurate method for the simultaneous and quantitative determination of these four major flavonoids by Ultra Performance Liquid Chromatography (UPLC) analysis was developed. This method should be appropriate for the quality assurance of COP. The UPLC separation was carried out using an octadecylsilane (ODS) Hypersil (2.1 mm × 250 mm, 1.9 μm) and a mobile phase composed of acetonitrile and 0.1% formic acid in water at a flow rate 0.44 mL/min and ultraviolet (UV) detection 254 nm. Gradient elution was employed. The method was precise, with relative standard deviation below 3.0% and showed excellent linearity (R^2^ > 0.999). The recoveries for the four flavonoids in COP were between 95.49%–106.23%. The average contents of LA, LE, CA and AP in different parts (flower, leave and stem) of COP were between 0.64–1.47 g/100 g, 0.66–0.89 g/100 g, 0.32–0.52 g/100 g and 0.16–0.18 g/100 g, respectively. The method was accurate and reproducible and it can provide a quantitative basis for quality control of COP.

## 1. Introduction

There is considerable recent evidence showing that free radicals induce oxidative damage to cause pathological effects on humans, including DNA damage, aging, and cancer [1,2]. Recently, there has been a global trend towards the using phenolic compounds extracted from fruits, vegetables, oilseeds, and herbal plants [3,4].

Dietary foods contain a variety of free radical scavenging antioxidants, such as phenolic compounds (tocopherols, flavonoids, and phenolic acids) [5]. Plant phenols have free radical scavenging properties due to their redox potential [6]. Phenolic compounds are commonly found in both edible and inedible plants, and have been reported to have multiple biological effects, including antioxidant activity. The antioxidant activity of phenolic compounds is mainly caused by their redox properties, which play an important role in adsorbing and neutralizing free radicals, by quenching singlet and triplet oxygen or by forming peroxides. Antioxidants are also of immense interest to health professionals, as they may help to protect the body against damage caused by reactive oxygen species (ROS) [7].

Dried Compositae (COP) flowers have traditionally been used in Korea for their anti-inflammatory and antioxidant activity. Plants from the family COP have been reported to have anti-inflammatory, antimicrobial, and antitumor properties [8,9,10,11]. Antioxidants are generally abundant in polyphenolic substances. The known major flavones present in COP plants are apigenin, acacetin, luteolin, diosmetin, eriodictyol, chlorogenic acid, and linarin [12,13]. Therefore, in the current study linarin, luteolin, chlorogenic acid, and apigenin were selected as the key compounds for extraction.

Method validation of analytical tests is conducted to ensure that the methodology is accurate, specific, reproducible, and robust over the specified range of analysis. Method validation provides an assurance of reliability during normal use, sometimes referred to as “the process of providing documented evidence that the method does what it is intended to do” [14]. Many validation methods including high-performance liquid chromatography (HPLC) [15], mass spectrometry (MS) [16], and capillary electrophoresis (CE) [17] have been used in the qualitative or quantitative analysis of natural extract, drug candidates and processed food. Despite the availability of various analytical techniques, currently there is an increasing demand for the fast and sensitive analysis of samples which could reduce costs and achieve high sample throughput. Among them, Ultra Performance Liquid Chromatography (UPLC) systems allow the use of small particle-packed columns with small diameter. The particles are designed to be able to resist high pressures, in contrast to conventional HPLC [18]. For this reason, UPLC can give improvements in speed, resolution, rapid and sensitivity of analysis, time savings, and solvent consumption compared to the previously used HPLC method [19]. Most of these advantages may be attributed to moving from HPLC to UPLC.

On the basis of the International Conference on Harmonization guidelines [20], an UPLC analytical method requires validation to confirm its linearity, recovery, and precision. In this study, a UPLC method for the quantification of flavonoid compounds from different parts of COP plant products was developed and validated for the first time. We show not only the validation of a reliable, fast, and easy methodology for quantification of flavonoid compounds, but in addition, the antioxidant capacity of the extracts was determined by 1,1-Diphenyl-2-picrylhydrazyl (DPPH) free radical-scavenging activity, and the contents of total polyphenols and total flavonoids were determined for different parts of COP plant products. Finally, the relationships between polyphenol and flavonoid contents and antioxidant activity were explored.

## 2. Results

### 2.1. Structural Determination of Isolate Compounds

**Figure 1:**
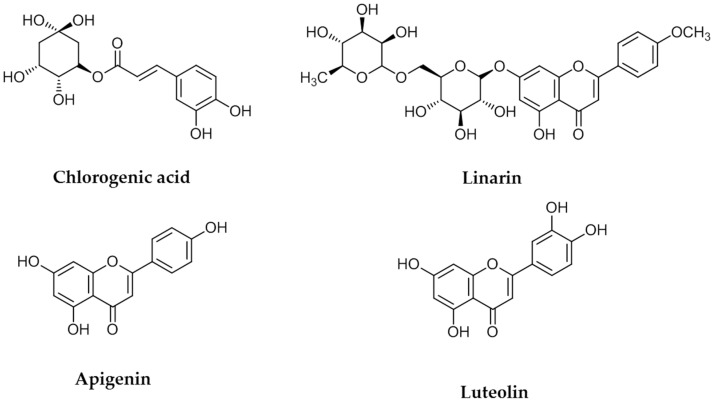
Chemical structures of chlorogenic acid, linarin, apigenin and luteolin.

The four flavonoids were separated from a 1 g sample of a primrose small Compositae (Kugya-sunjong, Table 1, Entry 13) methanol leaves extract by Sephadex LH-20 column chromatography to obtain chlorogenic acid (CA) (10.3 mg), luteolin (LE) (11.8 mg) apigenin (AP) (8.2 mg) and linarin (LA) (9.2 mg) (Figure 1). These compounds were identified by comparing their ^1^H- and ^13^C-NMR spectra and NMR correlation spectra such as correlation spectroscopy (COSY), heteronuclear multiple bond correlation (HMBC) and heteronuclear multiple quantum correlation (HMQC) with previously reported data [21,22].

### 2.2. Optimization of UPLC Conditions

The effectiveness of the UPLC separation was tested using the four flavonoids isolated from Entry 13. The gradient elution profile was optimized to obtain the highest resolution of four flavonoids and the shortest time of the analysis. Two solvents in a mobile phase 0.1% formic acid (FA) in water (solvent A) and acetonitrile (ACN) (solvent B), were selected and run according to the programmed gradient elution.

**Figure 2:**
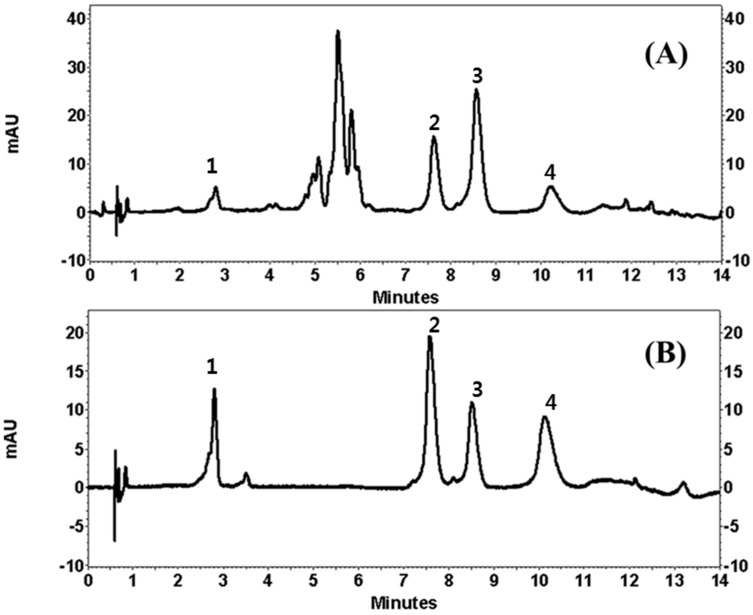
Ultra Performance Liquid Chromatography (UPLC) chromatogram of Compositae (Entry 13 in Table 1, (**A**)) and standard mixtures of the isolated compounds (1: chlorogenic acid; 2: linarin; 3: apigenin and 4: luteolin, (**B**)). mAU: miliabsorbance units.

Under the chromatographic conditions of the current experiment, the addition of 0.1% FA in water increased the resolution of the peaks. Unfortunately, neither isocratic elution nor gradient elution resulted in good chromatographic separation of LA and AP. Temperature was then used in this study. It was found that when the column temperature was maintained at 45 °C, it produced a good chromatographic peak. The wavelength for detection was tested at 254, 280, and 360 nm. The wavelength for detection was then set at 254 nm, which is where the four flavonoids showed the maximum absorption, as measured by a diode array detector (DAD). Therefore, the best resolution of all the peaks was obtained using a gradient of the mobile phase consisting of ACN and 0.1% FA in water within 14 min. The retention times of CA, LA, AP, and LE were 2.8, 7.7, 8.7, and 10.3 min (Figure 2).

### 2.3. Linearity

Serially diluted solutions of the four flavonoids prepared in the range of 1, 10, 25, 50 and 100 µg/mL were injected into the UPLC, and calibration curve equations were calculated. As shown in Table 2, Linearity (R^2^) showed a correlation higher than of 0.999, with the linear ranges also being determined. The detection and quantification limits for CA, LA, AP, and LT at the signal-to-noise ratio of the four flavonoids were 0.38, 0.13, 0.11, 0.26 µg/mL^−1^ and 1.15, 0.41, 0.35, 0.79 µg/mL^−1^, respectively.

### 2.4. Precision

Intra-day and inter-day variability was used to validate the precision of the UPLC method. To assess repeatability, four flavonoid standard solutions were injected three times at concentrations of 2.5, 10, and 100 µg/mL onto the UPLC system, and relative standard deviation (RSD) values were calculated for the retention time and peak area.

As shown in Table 3 and Table 4, the intra-day and inter-day peak area of the RSD were LA < 1.68%, LE < 1.87%, CA < 2.23%, and AP < 1.46%. The intra-day and inter-day retention time of the RSDs for the different compounds were <0.65% for LA, <1.12% for LE, <0.35% for CA, and <0.1% for AP, with an RSD of less than 3.0%.

### 2.5. Accuracy (Recovery)

A recovery test was used to determine accuracy. The methanol extract was spiked with four flavonoids to observe changes in the recovery rate (%). Accuracy was evaluated by measuring the mean recovery (%) of four flavonoids from the spiked extract solution versus the non-spiked extract sample. Each sample was analyzed three times, and the recovery rate was calculated by using the calibration curve obtained from the results of linearity test. The accuracy was determined for the different compounds, where LA had an accuracy of 99.12% ± 0.90%, LE 95.49% ± 0.23, CA 103.07% ± 0.36%, and AP 106.23% ± 0.33% (Table 5).

### 2.6. Quantification of the Four Compounds, Total Polyphenol Content, Total Flavonoid Content and Antioxidant Activity

Relatively high contents of LA, LE, and CA was observed from (Table 6): flowers of 10 and 18 COP (1.37 and 1.38 g/100 g), leaves of 6 and 11 COP (3.52 and 3.51 g/100 g), stems of 6 and 16 COP (1.53 and 1.27 g/100 g) of LA and flowers of 6, 9 and 10 COP (1.49, 1.55 and 1.64 g/100 g), leaves of 8, 11, 13 and 17 COP (1.36, 1.17, 1.31 and 1.39 g/100 g), of LE and flowers of 14 and 20 COP (1.02 and 1.02 g/100 g), leaves of 17 COP (2.55 g/100 g), of CA, respectively.

The leaf extracts contained more LA than the other plant parts. The following order of average LA content was observed: leaf (1.47 g/100 g) > stem (0.65 g/100 g) > flower (0.64 g/100 g). LE content showed the following order: flower (0.89 g/100 g) > leaf (0.66 g/100 g). LE was not observed in the stems. The average CA content had the following order: leaf (0.69 g/100 g) > stem (0.52 g/100 g) > flower (0.32 g/100 g). The average AP content was similar in both the leaves (0.18 g/100 g) and the flowers (0.16 g/100 g), but AP was not observed in the stem.

The content of the various compounds did not vary according to cultivar/breed. Total polyphenol content (TPC) and total flavonoid content (TFC) of different plant parts of the 20 COP are shown in Table 7. The TPC of 20 COP was determined using a linear gallic acid standard curve. The TPC of the 20 COP ranged from 0.31 to 6.78 g gallic acid equivalents (GAE)/100 g, with lower values being obtained from the stems, while the higher values were obtained from the flowers and leaves. The TFC of 20 COP was determined using a linear catechin standard. The TFC of the 20 COP ranged from 0.25 to 10.45 g catechin equivalents (CE)/100 g. The TFC of some leaves was higher than the content of flowers; however, most leaves had a lower content than the flowers. It has been previously reported that the TPC and TFC of *Chrysanthemum indicum* L. flower was 2.80 g/100 g and 1.89 g/100 g [23].

**Figure 3:**
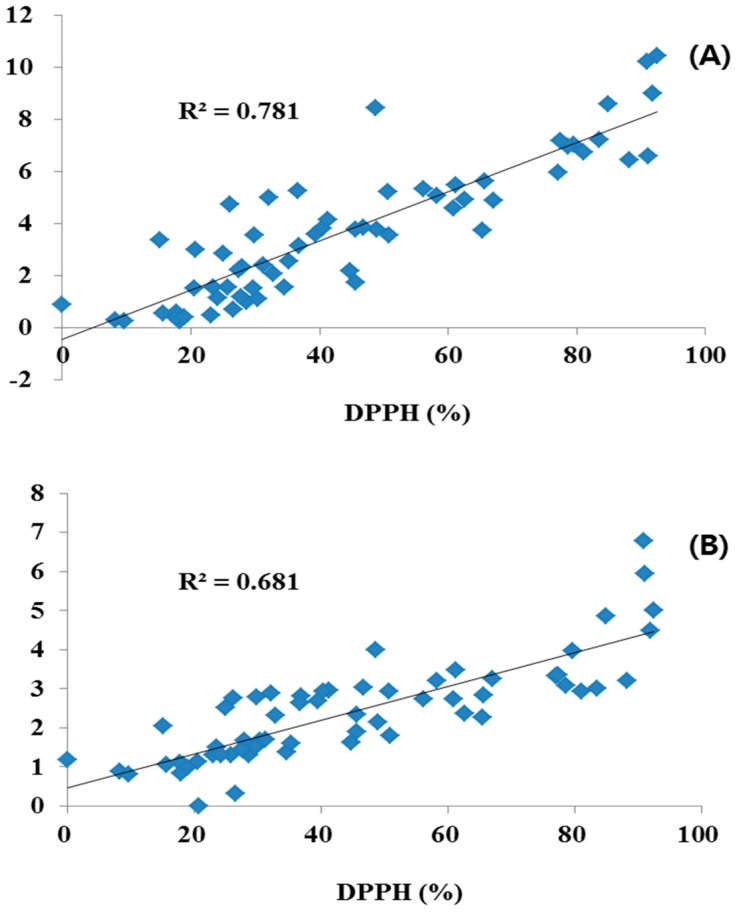
Correlation coefficients of antioxidant activity with total polyphenol (**A**) and total flavonoid content (**B**) in different parts of Compositae.

The different plant parts of the 20 COP had different antioxidant activity levels, as shown in Table 7. The methanol extract of the different plant parts of the 20 COP were initially evaluated for antioxidant activity at concentration of 1 mg/mL using the DPPH free radical scavenging test system. The plant extracts were derived from the flower, leaf and stem parts, which inhibited antioxidant activity by 8.17%–92.47%, 15.12%–91.17%, and 9.61%–88.2%. The flower parts of Red COP (Kugya-myungseong, Entry 6) exhibited the highest antioxidant activity: 92.47%. Antioxidant activity was significantly correlated with TPC and TFC (R^2^ = 0.681 and 0.781, respectively; Figure 3). Previous studies have reported strong relationships for antioxidant activity with TPC and TFC in several fruits, vegetables, and grain products [12]. The antioxidant activity was shown to be significantly correlated with TPC and TFC (R^2^ = 0.681 and 0.781, respectively).

## 3. Discussion

Quality assurance (QA) and quality control (QC) are very important and a suitable analytical method is needed to enable producers to check raw materials, processed foods and plant extracts. The developed UPLC method offers advantages over those previously reported using conventional liquid chromatography by showing a faster chromatographic total run time and better chromatographic performance. Since a high number of samples are needed for QA/QC analysis, the very fast UPLC separation combined with an adequate efficiency described in this work allows the application of the method for routine analysis. The applicability of our method was finally verified by using different parts of 20 COP. Extract stock solutions (10 μg/mL) were analyzed in order to evaluate peak separation and method suitability. Figure 2 showed the resulting chromatograms, where separation was highly satisfactory in all the samples.

The UPLC-DAD method developed in this study was applied, for the first time, to the quantitative analysis of the flavonoids identified in the different parts of COP methanol extracts. A weighed amount (10.0 mg) of dried methanol extract of the different parts of COP plant products was dissolved in 1.0 mL of methanol and filtered through a 0.45 μm filter before analysis. The external standard calibration curve of four flavonoids was generated by using five data points and injections were performed in triplicate for each concentration level (injection volume: 10 μL). In addition, the calibration curve was obtained by plotting the peak area of the compound at each level versus the concentration of the samples and the amount of the four flavonoids in sample extracts was determined by using the calibration curves of the compounds. Our analytical method was applied to simultaneous determination of the four flavonoids in the sample extracts. In Table 6, the flavonoid contents were expressed as g/100 g (dried material). The contents of LA, LE, CA and AP were in the following ranges: LA of 0.16–1.37 (flower), 0.10–3.52 (leave), 0.23–1.53 (stem) g/100 g; LE of 0.25–1.64 (flower), 0.23–1.39 (leave) g/100 g; CA of 0.14–1.02 (flower), 0.20–2.55 (leave), 0.13–0.55 (stem) g/100 g; AP of 0.02–0.27 (flower), 0.01–0.46 (leave) g/100 g dried material, respectively. 

The limit of detection (LOD) values of LA, LT, CA and AP were 0.13, 0.26, 0.38 and 0.11 μg/mL^−1^, respectively, and the limit of quantification (LOQ) values were 0.41 (LA), 0.79 (LT), 1.15 (CA) and 0.35 (AP) μg/mL^−1^ (Table 2). The LOD and LOQ values obtained for the standard compounds indicate a suitable sensitivity of the UPLC-DAD method proposed for the analysis of sample extracts. However, LE and AP contents were under the LOQ value in stem.

Leaves had a generally higher content of the four flavonoids (0.18–1.47 g/100 g) than flowers (0.16–0.89 g/100 g), whereas, stems had the lower contents of the four flavonoids (0.32–0.65 g/100 g) and LE and AP were not detected. On the other hand, the COP samples showed considerable differences in antioxidant activity over a 32.97%–54.20% range. We suggest that antioxidant activity was changed by the TPC and TFC. Among them, Entry 6 flowers showed strong antioxidant activity of 92.47% (TFC: 10.45 g/100 g; TPC: 5.00 g/100 g), but leaves and stems of Entry 6 showed lower activities with 36.65 (TFC: 5.26 g/100 g; TPC: 2.64 g/100 g) and 45.65% (TFC: 1.75 g/100 g; TPC: 1.91 g/100 g). In addition, Entry 17 leaves and stems had strong inhibition of 90.94% (TFC: 10.22 g/100 g; TPC: 6.78 g/100 g) and 88.20% (TFC: 6.45 g/100 g; TPC: 3.21 g/100 g), more than flowers 27.47% (TFC: 2.22 g/100 g; TPC: 1.46 g/100 g). Similarly, Entry 3, 10, 16 and 20 showed relatively high antioxidant activity; all of these samples also contained above average levels of TFC and TPC. The TFC-rich samples generally showed very strong activities with high TPC. Antioxidant activity was significantly correlated with TPC and TFC.

HPLC including the MS method allows the separation of most constituents, but there are several problems in applying this method to natural extracts: resolution is often incomplete, the analysis time too long and costly, and chromatographic performance quickly deteriorates [20]. Previously Zhang et al. reported on a rapid quantitative determination of andrographolide, neoandrographolide, 14-deoxyandrographolide and 14-deoxyl-11,12-didehydroandrographolide in *Andrographis paniculata* extract by reverse phase UPLC and gradient elution using ACN-water as mobile phase (0–2 min, 20%–25% ACN (A); 2–5 min, 25%–35% A; 5–7 min, 35% A; 7–10 min, 35%–55% A) at a flow rate of 0.5 min/mL, detecting wavelength at 220 nm [24]. Li et al. reported the development and validation of a UPLC method for the simultaneous quantification of five flavonoids (baicalin, wogonoside, baicalein, wogonin and oroxylin) in *Scutellariae Radix* [25]. Wang et al. also reported a UPLC quantification of LE, rutin, quercetin and betulinic acid in an extract of *Disporopsis pernyi* (Hua) Diels with more than 30 min of run time and a gradient elution with a mobile phase of ACN and water containing 0.1% FA in water and with a flow rate of 0.2 mL/min, with detection at 210, 254, and 280 nm [26]. In addition, Miranda et al. were to develop and validate a novel UPLC-DAD method for simultaneously quantifying chloroquine and primaquine in tablet formulations [27] and UPLC was completely validated and successfully applied to the pharmacokinetic and bioavailability study after sublingual vein and oral administration of corilagin to rats by Zheng et al. [19].

Our UPLC method represents an excellent technique for the simultaneous determination of LA, LE, CA and AP in COP with good sensitivity, accuracy, precision, and linearity. The method gives a good resolution of the four flavonoids with a short analysis run time (14 min). The UPLC method can be used as quality control of polyphenolic constituents in COP and can serve as a reference role for the determination of constituents in natural sources, drug candidate and pharmaceutical preparations.

## 4. Materials and Methods

### 4.1. General Experimental Procedures

UPLC was performed using an Accela UPLC system (Accela 1250, Thermo, Boston, MA, USA). ^1^H- and ^13^C-NMR spectra and correlation NMR spectra such as COSY, HMBC, HMQC, and distortionless enhancement by polarization transfer (DEPT) were obtained from an Avance DPX 400 (or 600) spectrometer (Bruker, Berlin, Germany) for identification of the isolated compounds. These were obtained at operating frequencies of 400 MHz (^1^H) and 100 (or 150) MHz (^13^C) with CD_3_OD, (CD_3_)_2_SO, (CD_3_)_2_CO, or D_2_O and tetramethylsilane (TMS) was used as an internal standard. DPPH was obtained from Sigma Chemical Co. (St. Louis, MO, USA). TPC and TFC were determined using the Folin-Ciocalteu phenol reagent and aluminum chloride colorimetric from Sigma-Aldrich Co. All solvents used in the analysis were HPLC grade obtained from J.T. Baker (Phillipsburg, NJ, USA).

### 4.2. Extraction and Isolation

Twenty COP plants (Table 1) were supplied by the “Kugya Farm” (Chuncheon, Korea). There were 11 variants of *Chrysanthemum morifolium*, six variants of *Chrysanthemum indicum,* one *Aster sphathulifolius* Maxim, one *Chrysanthemum zawadskii* var. *latilobum*, and one *Dendranthema makinoi*. The fresh COP plants were dried at 45 °C in a drying oven and then stored at room temperature. The dried COP plants were then ground to less than 0.5-mm pieces by using a grinder (JL-500, Joy-life, Seoul, Korea). Different plant parts of the dried COP (20 g) were extracted twice with methylene chloride (200 mL) at room temperature, using a shaker for 24 h. Then, the residue was mixed for 24 h with methanol (200 mL × 2). The filtrate was concentrated until dry under reduced pressure on a rotary evaporator at 40 °C. The CA, LE, AP and LA from leaves of Entry 13 methanol extract were isolated by column chromatography. Briefly, Entry 13 (1.0 g) was dissolved in methanol and loaded onto a Sephadex LH-20 column and partitioned to obtain seven fractions. CA and LE were directly obtained from fraction 1 and fraction 4, respectively. Fraction 5 was subsequently separated by a silica-gel column eluted by a solvent mixture of methylene chloride and methanol (from 20:0 to 1:1, *v*/*v*) to obtain AP. LA was purified from fraction 7 on a Sephadex LH-20 column eluted with 70% methanol.

### 4.3. UPLC Analysis

The samples were analyzed on a Thermo UPLC system (Accela 1250, Thermo). UPLC separation was accomplished on an octadecylsilane (ODS) Hypersil column (2.1 mm × 250 mm, 1.9 μm, Thermo). The mobile phase, consisting of ACN and 0.1% formic acid in water, was used at a flow rate of 0.44 mL/min. The gradient elution program was: 5%–15% ACN (B) (0–1 min), 15% B (1–4 min), 15%–25% B (4–5 min), 25%–40% B (5–6 min), 40%–55% B (6–8 min), 55%–100% B (8–9 min), 100% B (9–11 min), 100%–5% B (11–12 min), and 5% B (12–14 min). The injection volume was 5 μL, and the detection wavelength was 254 nm. The column temperature was maintained at 45 °C using a temperature controller.

### 4.4. Method Validation of Quantitative Analysis

Following the specifications of the International Conference on Harmonization guidelines, the analytical method was validated by determining the linearity, LOD, and LOQ, precision, repeatability, and accuracy for each analyte.

### 4.5. Linearity

Linearity was examined using four flavonoid solutions. The four flavonoids were dissolved in methanol to prepare the standard solutions. Five different concentration of standard solutions dissolved in methanol were prepared at a range of 1, 10, 25, 50 and 100 μg/mL by the serial dilution method. The linearity of the calibration curves was determined by plotting the mean peak area (*y*-axis) versus concentration (*x*-axis) for each analyte in this range.

### 4.6. Limit of Detection and Limit of Quantification

After injecting an aliquot (10 µL) of the serial dilutions of 5 individual standard solutions, the LODs and LOQs under the selected UPLC method were determined at signal to noise (S/N) ratios of 3 and 10, respectively.

### 4.7. Precision and Accuracy

Intra- and inter-day variability of the COP extracts were measured to validate the precision. Intra-day variability was determined by analyzing the samples within 24 h. The solutions were injected 3 times, and the RSD value was calculated for the concentration of each analyte in the extract, and was assumed to represent the measure of precision. Each sample was injected three times a day on three consecutive days to assess inter-day variability. Accuracy was evaluated in a recovery test by calculating the mean recovery (%) of the four flavonoids from a spiked extract solution versus an unspiked extract sample.

### 4.8. DPPH Assay

The stable free radical was used to determine the free radical-scavenging activity of the extracts [28]. Briefly, a 0.32-mM solution of DPPH in methanol was prepared, and then 180 µL of this solution was mixed with 30 µL of each sample (crude extract) at concentrations of 1 mg/mL in methanol. After 15 min incubation in the dark, the decrease in the absorbance of the solution was measured at 570 nm on a microplate reader (EL800 Universal Microplate reader, Bio-Tek Instruments, Inc., Winooski, VT, USA). DPPH inhibitory activity was expressed as the percentage inhibition (%) of DPPH in the above assay system, and was calculated as (1 − B/A) × 100, where A and B are the activities of DPPH without and with the test material, respectively.

### 4.9. Determination of Total Polyphenol Content

The TPC content of different parts of COP plants were determined by using the Folin-Ciocalteu reagent, according to the association of official analytical chemists (AOAC) Folin-Ciocalteu method [29]. An aliquot (25 µL) of samples (1 mg/mL) or standard solution of gallic acid (0.05, 0.1, 0.25, 0.5 and 1 mg/mL) was added to a 1.5 mL test tube, containing 75 µL of Folin-Ciocalteu reagent. After 5 min, 200 µL of 7% Na_2_CO_3_ solution was added to the mixture. After 5 min, 700 µL of distilled water was added, and placed in the dark at room temperature for 60 min. The absorbance of all samples was measured at 750 nm using an EL 800 Universal Microplate Reader (Bio-Tek Instruments, Inc.). The TPC of the COP extracts was expressed as g GAE/100 g dry weight. The reported data are the combined results of the 3 replications.

### 4.10. Determination of Total Flavonoid Content

TFC of different parts of COP plants was determined by using the aluminum chloride colorimetric method [30]. An aliquot (100 µL) of samples (1 mg/mL) or standard solution of catechin (0.05, 0.1, 0.25, 0.5, and 1 mg/mL) was added to an 1.5 mL test tube, containing 400 µL of D_2_O, with 30 µL of 5% NaNO_2_ being added to this mixture. After 5 min, 30 µL of 10% AlCl_3_ solution was added to the mixture. The mixture was allowed to stand at room temperature for 6 min. 200 µL of 1 M NaOH was added, and the total volume was made up to 1 mL with D_2_O. The absorbance of all samples was measured at 510 nm using an EL 800 Universal Microplate Reader (Bio-Tek Instruments, Inc.). The TFC of the COP extracts was expressed as g CE/100 g dry weight. The reported data are the combined results of three replications.

## 5. Conclusions

Four flavonoids which were isolated from COP, have been reported to exert many pharmacological activities. A simple, accurate and rapid UPLC coupled to DAD method has been developed to quantify these four flavonoids in COP. The method was successfully validated. To the best of our knowledge, it is the first time that a UPLC gradient method has been applied to the simultaneous determination of four flavonoids in the COP. The developed method offers advantages over those previously reported using conventional liquid chromatography by showing a faster chromatographic total run time (14 min) and better chromatographic performance: Separation was performed on a ODS Hypersil (2.1 mm × 250 mm, 1.9 μm) column by using a mobile phase of ACN and water with 0.1% formic acid (*v*/*v*), at a flow rate 0.44 mL/min. The method was validated in terms of selectivity, linearity, accuracy, precision and recovery. Good linearity was observed over the investigated concentration range (1–100 μg/mL), with correlation coefficient values greater than 0.99. The intra- and inter-day precisions over the concentration range were between 1.46%–2.23% (RSD), and the accuracy was between 95.49%–106.23%. In addition, LA, LE, CA and AP in different parts of COP were analyzed between 0.64–1.47 g/100 g, 0.66–0.89 g/100 g, 0.32–0.52 g/100 g and 0.16–0.18 g/100 g, respectively. Our results suggested that since a high number of samples are needed for quality control analysis, the very fast UPLC separation combined with an adequate efficiency described in this work allows the application of the method for routine and QA/QC analysis.

## Figures and Tables

**Table 1:** List of Compositae family samples.

Entry	Plant Species	
	Name	Scientific Name
1	Double flower Compositae (Kugya-baekcheon)	*Chrysanthemum morifolium* variants
2	Red Korean Compositae (Kugya-seonnyeo)	*Chrysanthemum morifolium* variants
3	Dark red Compositae (Kugya-jinju)	*Chrysanthemum morifolium* variants
4	Pink Compositae (Kugya-dowon)	*Chrysanthemum morifolium* variants
5	Yellow short Compositae (Kugya-hana)	*Chrysanthemum morifolium* variants
6	Red Compositae (Kugya-myungseong)	*Chrysanthemum morifolium* variants
7	Yellow small Compositae (Kugya-baram)	*Chrysanthemum morifolium* variants
8	White double Compositae (Kugya-cheonsa)	*Chrysanthemum morifolium* variants
9	Dark yellow Compositae (Kugya-gyeongjin)	*Chrysanthemum morifolium* variants
10	White short Compositae (Kugya-somang)	*Chrysanthemum morifolium* variants
11	Yellow Compositae (Kugya-kughang)	*Chrysanthemum morifolium* variants
12	White Compositae (Kugya-sinsun)	*Chrysanthemum indicum* variants
13	Primrose small Compositae (Kugya-sunjong)	*Chrysanthemum indicum* variants
14	Primrose short Compositae (Kugya-gamhea)	*Chrysanthemum indicum* variants
15	Yellow large Compositae (Kugya-gamthae)	*Chrysanthemum indicum* variants
16	White small Compositae (Kugya-sulwha)	*Chrysanthemum indicum* variants
17	Primrose Compositae (Kugya-sunjeong)	*Chrysanthemum indicum* variants
18	Yellow lanugo Compositae (Kugya-gumi)	*Chrysanthemum zawadskii* var. *latilobum*
19	White lanugo Compositae (Kugya-baekhae)	*Aster sphathulifolius Maxim*
20	Makino Compositae (Kugya-makino)	*Dendranthema makinoi (Matsum)*

**Table 2:** Statistical analysis for the calibration curves of four flavonoids (*n* = 3).

Compound	Slope	Intercept	R^2 a^	LOD ^b^	LOQ ^c^
Linarin	4600.44 ± 73.86	−9.71 ± 1.89	0.999	0.13	0.41
Luteolin	6829.50 ± 185.08	−107.54 ± 54.17	0.999	0.26	0.79
Chlorogenic acid	3400.03 ± 51.41	−5.01 ± 3.92	0.999	0.38	1.15
Apigenin	5614.93 ± 56.57	16.26 ± 2.01	0.999	0.11	0.35

^a^ R^2^, correlation coefficient for the 5 data points in the calibration curves (*n* = 3); ^b^ LOD, limit of detection (µg/mL^−1^, Signal to noise (S/N) = 3); **^c^** LOQ, limit of quantification (µg/mL^−1^, S/N = 10).

**Table 3:** Intra-day precision data for the retention time and peak area of four flavonoids.

Concentration (µg/mL)	Intra-day Precision (*n =* 3)							
	Linarin		Luteolin		Chlorogenic Acid		Apigenin	
	Rt ^a^	Area ^b^	Rt	Area	Rt	Area	Rt	Area
100	0.07	1.56	0.20	0.60	0.25	1.70	0.08	0.94
10	0.23	1.47	0.13	2.15	0.24	0.45	0.04	1.53
2.5	0.01	1.68	0.24	1.87	0.38	2.23	0.05	1.46

**^a^** Relative standard deviation of retention time (Rt) (% RSD); **^b^** Relative standard deviation of peak area (% RSD).

**Table 4:** Inter-day precision data for the retention times and peak area of four flavonoids.

Concentration (µg/mL)	Inter-Day Precision (*n =* 3)							
	Linarin		Luteolin		Chlorogenic Acid		Apigenin	
	Rt ^a^	Area ^b^	Rt	Area	Rt	Area	Rt	Area
100	0.65	0.29	1.12	0.68	0.35	0.86	0.10	1.77
10	0.61	1.47	0.12	2.21	0.29	1.20	0.03	1.38
2.5	0.06	1.38	0.33	2.34	0.09	2.04	0.08	2.05

^a^ Relative standard deviation of retention time (% RSD); ^b^ Relative standard deviation of peak area (% RSD).

**Table 5:** Recovery data of four flavonoids.

Compounds	Original Amount (µg)	Spiked Amount (µg)	Determined Amount (µg)	Recovery (%, Mean ± RSD, *n =* 3)
Linarin	13.91	9.80	23.49	99.12 ± 0.90
Luteolin	6.29	9.80	15.37	95.49 ± 0.23
Chlorogenic acid	4.19	9.50	14.12	103.07 ± 0.36
Apigenin	3.02	9.70	13.49	106.23 ± 0.33

**Table 6:** Linarin, luteolin, chlorogenic acid, and apigenin content in different parts of Compositae (g/100 g).

Entry	Flower (*n =* 3)				Leave (*n =* 3)				Stem (*n* = 3)			
	Linarin	Luteolin	Chlorogenic Acid	Apigenin	Linarin	Luteolin	Chlorogenic Acid	Apigenin	Linarin	Luteolin	Chlorogenic Acid	Apigenin
1	0.24 ± 0.008	0.25 ± 0.011	0.29 ± 0.059	-	1.32 ± 0.051	0.34 ± 0.001	0.37 ± 0.022	0.01 ± 0.010	0.77 ± 0.027	-	0.25 ± 0.012	-
2	0.67 ± 0.118	-	-	-	1.87 ± 0.002	0.45 ± 0.013	0.26 ± 0.044	0.26 ± 0.016	0.66 ± 0.119	-	-	-
3	0.32 ± 0.035	-	0.62 ± 0.081	-	0.55 ± 0.092	0.78 ± 0.012	0.24 ± 0.008	-	0.40 ± 0.146	-	0.15 ± 0.007	-
4	-	0.54 ± 0.051	0.14 ± 0.033	0.02 ± 0.012	0.10 ± 0.004	0.32 ± 0.002	0.49 ± 0.140	0.13 ± 0.034	-	-	0.23 ± 0.001	-
5	0.08 ± 0.007	0.52 ± 0.027	0.58 ± 0.096	-	0.29 ± 0.029	0.86 ± 0.110	0.16 ± 0.013	-	-	-	-	-
6	1.10 ± 0.086	1.49 ± 0.010	0.78 ± 0.018	-	3.52 ± 0.077	0.34 ± 0.005	0.30 ± 0.022	-	1.53 ± 0.641	-	0.19 ± 0.002	-
7	0.48 ± 0.077	0.39 ± 0.041	0.27 ± 0.011	-	3.33 ± 0.297	0.34 ± 0.012	0.77 ± 0.017	0.11 ± 0.008	0.64 ± 0.027	-	0.42 ± 0.005	-
8	0.17 ± 0.001	0.43 ± 0.013	-	-	2.00 ± 0.061	1.36 ± 0.087	0.43 ± 0.020	-	0.23 ± 0.038	-	-	-
9	-	1.55 ± 0.057	-	-	0.17 ± 0.007	0.32 ± 0.001	-	-	-	-	-	-
10	1.37 ± 0.002	1.64 ± 0.069	-	0.21 ± 0.001	1.92 ± 0.119	0.73 ± 0.045	-	0.16 ± 0.004	0.56 ± 0.109	-	-	-
11	0.59 ± 0.065	0.95 ± 0.204	-	-	3.51 ± 0.061	1.17 ± 0.088	0.20 ± 0.005	0.15 ± 0.006	0.74 ± 0.028	-	-	-
12	0.39 ± 0.071	0.82 ± 0.100	0.52 ± 0.057	-	0.54 ± 0.030	0.30 ± 0.004	0.25 ± 0.002	-	0.23 ± 0.141	-	-	-
13	0.25 ± 0.042	0.40 ± 0.031	0.19 ± 0.010	-	0.80 ± 0.031	1.31 ± 0.051	0.29 ± 0.035	0.46 ± 0.002	0.44 ± 0.078	-	0.13 ± 0.014	-
14	1.34 ± 0.220	0.41 ± 0.010	1.02 ± 0.072	-	2.10 ± 0.043	-	2.95 ± 0.014	-	0.89 ± 0.263	-	0.33 ± 0.005	-
15	0.16 ± 0.025	-	-	-	0.58 ± 0.045	-	-	-	0.15 ± 0.033	-	-	-
16	1.29 ± 0.076	0.78 ± 0.026	-	0.12 ± 0.013	0.89 ± 0.039	0.23 ± 0.003	0.69 ± 0.038	-	1.27 ± 0.252	-	0.35 ± 0.020	-
17	0.46 ± 0.036	0.37 ± 0.005	-	-	2.62 ± 0.102	1.39 ± 0.034	2.55 ± 0.076	-	0.73 ± 0.185	-	0.87 ± 0.023	-
18	1.38 ± 0.277	0.89 ± 0.171	0.34 ± 0.092	0.27 ± 0.099	0.31 ± 0.012	0.35 ± 0.011	0.28 ± 0.009	-	0.49 ± 0.076	-	0.13 ± 0.001	-
19	-	-	0.58 ± 0.049	0.18 ± 0.014	-	-	0.41 ± 0.023	-	-	-	0.22 ± 0.002	-
20	-	0.73 ± 0.100	1.02 ± 0.072	-	-	-	1.13 ± 0.057	-	-	-	0.55 ± 0.004	-
Average	0.64	0.89	0.52	0.16	1.47	0.66	0.69	0.18	0.65	-	0.32	-

**Table 7:** Total polyphenol content (TPC, gallic acid equivalents (GAE) g/100 g), total flavonoid content (TFC, catechin equivalents (CE) g/100 g), and the 1,1-Diphenyl-2-picrylhydrazyl (DPPH) scavenging activity (%, 1 mg/mL) in different parts of Compositae.

Entry	Flower (*n* = 3)			Leave (*n* = 3)			Stem (*n* = 3)		
	DPPH (%)	TFC	TPC	DPPH (%)	TFC	TPC	DPPH (%)	TFC	TPC
1	20.47 ± 2.66	1.52 ± 0.06	1.13 ± 0.08	39.47 ± 0.42	3.58 ± 0.23	2.68 ± 0.18	67.07 ± 2.00	4.89 ± 0.04	3.25 ± 0.29
2	8.17 ± 0.75	0.31 ± 0.04	0.88 ± 0.06	25.04 ± 1.91	2.83 ± 0.16	2.51 ± 0.15	23.04 ± 2.52	0.47 ± 0.10	1.32 ± 0.09
3	81.17 ± 0.17	6.73 ± 0.13	2.93 ± 0.04	36.82 ± 1.83	3.13 ± 0.04	2.81 ± 0.26	30.32 ± 0.83	1.10 ± 0.14	1.67 ± 0.13
4	48.88 ± 1.58	3.78 ± 0.31	2.14 ± 0.08	50.65 ± 0.08	5.21 ± 0.47	2.94 ± 0.24	25.72 ± 1.12	1.55 ± 0.07	1.32 ± 0.15
5	62.64 ± 3.58	4.91 ± 0.30	2.36 ± 0.19	29.82 ± 2.75	3.55 ± 0.04	2.78 ± 0.30	17.67 ± 1.78	0.60 ± 0.03	1.11 ± 0.12
6	92.47 ± 0.67	10.45 ± 0.41	5.00 ± 0.97	36.65 ± 1.25	5.26 ± 0.45	2.64 ± 0.26	45.65 ± 2.77	1.75 ± 0.04	1.91 ± 0.04
7	35.29 ± 3.66	2.54 ± 0.34	1.60 ± 0.17	48.70 ± 0.83	4.42 ± 0.34	3.99 ± 0.02	65.44 ± 0.32	3.72 ± 0.01	2.27 ± 0.25
8	31.29 ± 2.00	2.42 ± 0.23	1.70 ± 0.24	46.76 ± 1.41	3.85 ± 0.21	3.04 ± 0.22	18.94 ± 2.32	0.40 ± 0.08	0.99 ± 0.08
9	56.17 ± 0.58	5.31 ± 0.21	2.75 ± 0.44	32.76 ± 0.58	2.08 ± 0.03	2.31 ± 0.18	15.62 ± 0.25	0.56 ± 0.03	1.05 ± 0.13
10	77.47 ± 1.25	7.16 ± 0.34	3.35 ± 0.27	26.11 ± 1.00	4.72 ± 0.37	2.76 ± 0.29	26.50 ± 1.27	0.71 ± 0.04	0.31 ± 0.34
11	45.58 ± 0.25	3.78 ± 0.37	2.35 ± 0.37	40.35 ± 1.00	3.80 ± 0.11	2.93 ± 0.27	24.17 ± 0.59	1.14 ± 0.03	1.32 ± 0.14
12	60.94 ± 1.00	4.58 ± 0.11	2.73 ± 0.25	15.12 ± 1.08	3.38 ± 0.08	2.04 ± 0.17	18.31 ± 1.00	0.27 ± 0.04	1.06 ± 0.07
13	27.88 ± 0.83	2.32 ± 0.11	1.69 ± 0.15	41.23 ± 0.08	4.13 ± 0.24	2.96 ± 0.32	28.69 ± 2.12	1.01 ± 0.13	1.30 ± 0.13
14	78.71 ± 2.99	6.94 ± 0.28	3.08 ± 0.34	91.17 ± 0.10	6.59 ± 0.01	5.95 ± 0.33	29.61 ± 1.84	1.53 ± 0.10	1.50 ± 0.11
15	23.47 ± 2.25	1.54 ± 0.25	1.51 ± 0.15	19.64 ± 0.72	0.87 ± 0.07	1.19 ± 0.16	9.61 ± 3.11	0.25 ± 0.04	0.82 ± 0.08
16	83.52 ± 0.83	7.23 ± 0.40	3.02 ± 0.52	61.29 ± 0.67	5.49 ± 0.52	3.47 ± 0.24	34.63 ± 2.16	1.55 ± 0.13	1.37 ± 0.07
17	27.47 ± 0.75	2.22 ± 0.34	1.46 ± 0.12	90.94 ± 0.56	10.22 ± 0.20	6.78 ± 0.69	88.20 ± 0.39	6.45 ± 0.47	3.21 ± 0.18
18	65.64 ± 0.83	5.62 ± 0.31	2.84 ± 0.35	32.05 ± 0.75	4.99 ± 0.10	2.88 ± 0.01	27.77 ± 1.81	1.17 ± 0.13	1.44 ± 0.11
19	77.11 ± 1.41	5.94 ± 0.28	3.34 ± 0.39	20.76 ± 3.08	3.01 ± 0.24	2.51 ± 0.08	17.81 ± 1.92	0.38 ± 0.08	0.83 ± 0.23
20	79.64 ± 1.83	7.02 ± 0.23	3.96 ± 0.33	58.35 ± 2.33	5.06 ± 0.03	3.21 ± 0.25	44.73 ± 1.80	2.17 ± 0.07	1.64 ± 0.16
Average	54.20	4.61	2.49	42.18	4.51	3.12	32.97	1.58	1.48
